# A patient-derived stem cell model of hereditary spastic paraplegia with *SPAST* mutations

**DOI:** 10.1242/dmm.023002

**Published:** 2015-10-01

**Authors:** Greger Abrahamsen, Yongjun Fan, Nicholas Matigian, Gautam Wali, Bernadette Bellette, Ratneswary Sutharsan, Jyothy Raju, Stephen A. Wood, David Veivers, Carolyn M. Sue, Alan Mackay-Sim

There was an error published in *Dis. Model. Mech.* **6**, 489-502.

Due to a technical error with the image analysis software, the published speed unit of peroxisome movement is incorrect. The stated peroxisome speed unit (µm/second) should be changed to µm/2 seconds in two different places in the paper: (1) twice in the Results section ‘Dynamics of peroxisome movement’, and (2) the *x*-axis of Fig. 5 (the legend remains the same). The correct text and figure is shown below. This change does not alter any conclusions of the paper, which was a comparison of cells from patients and controls.

(1) The mean peroxisome speed in patient cells was 93% slower than in control cells (control, 0.172±0.001 μm/2 seconds; patient, 0.160±0.001 μm/2 seconds; *t*=9.19, d.f.= 24,398, *P*<0.0001).

**Fig. 5.**
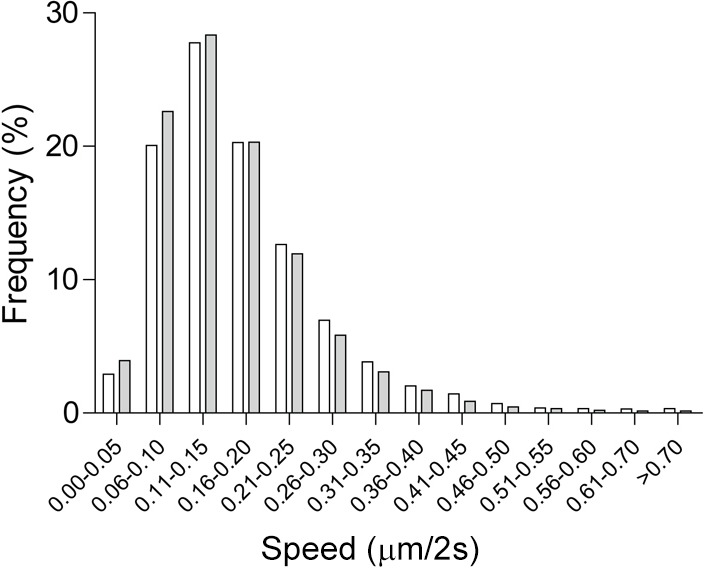
(2) **Fig. 5. Peroxisome speeds were slower in patient and control cells.** Frequency distributions of peroxisomes in different speed classes are shown for control cells (open bars; *n*=7 individuals, 10 cells per individual) and patient cells (filled bars; *n*=6 individuals, 10 cells per individual). Peroxisome speeds were quantified every 2 seconds for 2 minutes and grouped in speed classes expressed as the percentage of peroxisomes in each speed class as a percentage of the total number of peroxisomes for each group (control cells, *n*=13,871 peroxisomes; patient cells, 10,529 peroxisomes).

The authors apologise to the readers for any confusion that this error might have caused.

